# Using Community-Based Research Strategies in Age-Friendly Communities to Solve Mobility Challenges

**DOI:** 10.1093/geroni/igaa057.2462

**Published:** 2020-12-16

**Authors:** Holly Dabelko-Schoeny, Noelle Fields, Katie White, Marisa Sheldon, Sarah Robinson, Ian Murphy, Claire Jennings

**Affiliations:** 1 The Ohio State University, Columbus, Ohio, United States; 2 The University of Texas at Arlington, Arlington, Texas, United States; 3 Age Friendly Columbus, Columbus, Ohio, United States; 4 University of Texas at Arlington, Arlington, Texas, United States

## Abstract

Aging is linked to an increased risk of disability. Disabilities that limit major life activities such as seeing, walking, and motor skills impact a person’s ability to drive a car. Low utilization of alternative transportation by older adults may put them at risk for social isolation. The purpose of this paper is to illustrate how community-based participatory research (CBPR) was used to engage older residents in the development of alternative transportation options in a metropolitan county in the Midwestern U.S. Older residents worked as co-investigators to develop, use and evaluate alternative transportation options including walking, biking, fixed route busing, senior circulator, ride sharing, and transit training. Data were collected through mapping the built environment, an electronic daily transportation diary app called “MyAmble” on tablets, walk audits and focus groups. CBPR approaches led by interdisciplinary teams resulted in community engagement and more equitable strategies for transportation planning and utilization.

